# Resilient futures of a small island: A participatory approach in Tenerife (Canary Islands) to address climate change

**DOI:** 10.1016/j.envsci.2017.11.008

**Published:** 2018-02

**Authors:** Yeray Hernandez, Ângela Guimarães Pereira, Paulo Barbosa

**Affiliations:** a European Commission, Joint Research Centre (JRC), Directorate for Space, Security and Migration, Ispra, Italy; b European Commission, Joint Research Centre (JRC), Ispra, Italy

**Keywords:** Adaptation, Climate change, Citizen engagement, Stakeholder engagement, Focus group, Resilience

## Abstract

Adaptation to climate change has been considered to be crucial to current societies, especially for small islands. In this paper the case of Tenerife (in the Canary Islands) is analysed. Tenerife is a small island located northwest of the African continent, in the Atlantic Ocean. Tenerife presents a high vulnerability to heatwaves and Saharan dust events as a consequence of its closeness to the Saharan desert. In fact, increasing frequency of heatwaves and Saharan dust events has been reported and could worsen in the future due to global warming. An exploration of adaptation strategies to an increase of the frequency and intensity of these phenomena is therefore needed. Different social actors have been engaged in a participatory process aiming at exploring pathways for adaptation to extreme weather events. Resilience was argued as the relevant framing to address those hazards. Four focus group sessions were carried out in order to explore key transformative elements necessary to make resilient futures for Tenerife. The results highlight the need for broader climate-based policies across all sectors to assure that the island becomes resilient to climatic and non-climatic shocks.

## Highlights

• Stakeholders and citizens were engaged in a participatory process.
• Climate change adaptation frame is too narrow for small island contexts.
• Resilience is a helpful framing to discuss strategies to tame hazardous events.
• Resilience was defined as energy, food and water sovereignty.

## 1 Introduction

Small islands are already being affected by climate change (CC): morbidity and mortality are a consequence of extreme weather events, as well as vector- and food- and water-borne diseases (Nurse et al., 2014). Those extreme weather events refer to tropical cyclones, storm surges, flooding, and droughts leading to effects on human health, including drowning, injuries, disease transmission, and health effects derived from poor water quality (Nurse et al., *Op.cit.*).

Heatwaves are also seen as an increasingly dangerous climatic hazard since both, warm days and nights have increased globally (EEA, 2016, IPCC, 2014). Heatwaves are expected to occur more often and last longer (IPCC, *Op. cit.*), and Southern Europe is expected to be the most affected area in terms of hot weather, experiencing the highest heatwave exposure (IPCC, *Op. cit.*).

The work presented here refers to a case study for the Tenerife Island (Canary Islands) focused on heatwaves and strategies to cope with plausible yet uncertain developments. As heatwaves’ effects are sometimes connected to Saharan dust events and local air pollution episodes, these will also be considered. This paper describes an engagement activity carried out in Tenerife that sought to get insights from different actors about possible CC adaptation strategies and their implementation. The main objective of this work was to provide insights from different actors about the institutional and societal desirability and feasibility of adaptation strategies to CC in an island like Tenerife. We shall argue that establishing what and for whom is desirable and feasible requires the involvement of the “extended peer community" (Funtowicz and Ravetz, 1993). Four focus groups were carried out across the island with different social actors, including stakeholders and citizens. The ultimate aim of those group discussions was to develop visions of resilience and CC adaptation of Tenerife having heatwaves as the starting point. We first introduce the case study, followed by a review of cases on adaptation and resilience. The participatory methodology is then introduced followed by a discussion of the results, where we provide insights from the participatory process into strategies, practices, and institutional arrangements to address CC adaptation in small islands.

## 2 Heatwaves in Tenerife

Heatwaves are not only impacting on human health, but also on other species and ecosystems, as well as critical infrastructure, such as hospitals, transport and energy infrastructure, as a consequence of material overheating (IPCC, 2014). For instance, the heatwave of 2003 occurred in Western and central Europe produced damages to road and rail transport systems, interrupting energy supply, and increasing waterway transport prices as a consequence of low water levels associated to hydrological drought (IPCC, *Op.cit.*).

The hottest summer in Europe in the last 500 years was observed in 2003, leading to high death rates that caused 70,000 deaths in 12 European countries (EEA, 2015). By 2050, heatwaves are expected to produce 120,000 additional deaths annually in the European Union, especially among elderly people (EEA, 2015). A recent study from Forzieri et al. (2017) mentioned heatwaves as the most lethal weather-related hazard in Europe leading to an increased mortality rate of 5400% by the end of the century in a “business as usual” scenario and no climate adaptation measures. Morbidity is also co-related to warm spells. In fact, it is known that skin eruptions, fatigue, cramps, syncope, heat exhaustion, and heatstroke might occur as a consequence of heat exposure (WHO, 2004). The importance of these phenomena for the Canary Islands is also quite prominent as we will see.

Climate in the Canary Islands is mild, due to the influence of the NNE trade winds and the cool waters of the subtropical North Atlantic. These conditions prevent these Islands to suffer from the extreme weather conditions of the nearby Sahara desert, the largest and among the hottest deserts in the world. Episodically, cool trade wind weakens and easterly Saharan air reaches the Canaries. These Saharan air masses may prompt high temperatures, drops in relative humidity down to ∼15% (Dorta, 1991) and the presence of suspended desert dust. These heatwaves are mainly produced between spring and autumn (Dorta, 2007), usually reaching temperatures of 44–45 ° Celsius (Dorta, 1991); whereas night heat events reach maximums between 26 and 30 ° Celsius (Dorta, 2007).

The most dangerous documented heatwaves took place in August 1990 and July 2004 (Dorta, 2007). According to Alonso Pérez (2007), such episodes might have increased in intensity and frequency in the Canary Islands since 1970. In fact, the average number of heatwaves has quadrupled since 1994 and among the 10 strongest heatwaves recorded over the whole period, 5 have been detected during 2004–2007 (Sanz et al., 2007). Other authors also mention that a general rise of temperatures is expected for the Canary Islands in the future, intensified in upper parts of the islands (Martín et al., 2012). The last heatwaves registered in the Canary Islands have left 13 premature deaths, more than any other meteorological hazard (Dorta, 2007).

Other climate hazards that might be related to the effects of heatwaves are dust events. There are two dust seasons in the Canary Islands, one in the winter and another in the summer. In the winter dust season (November – March), Saharan dust events are associated with the easterly winds prompted by the occurrence of high pressure expanding from the North Atlantic over Western Europe and North Africa (Alonso-Pérez et al., 2011). These events may induce extremely high concentrations at ground level (up to 2000 μg/m^3^ have been recorded) and are not necessarily associated with high temperatures. Dust concentrations have duplicated since 1980.

However, in the summer season (July and August) dust events are associated with the circulation of the dusty Saharan Air Layer (SAL) – i.e. the hot and dry airstream that expands from North Africa to the Americas – over the Canary Islands. The SAL results in hot, dry and dusty air between 500 m.a.s.l. and 5 km.a.s.l. above the Canary Islands, whereas trade winds prevail below. Recent long-term analysis (1941–2013) of aerosol optical depth retrievals shows that there is an important multidecadal variability in summer dust export connected to the North African Dipol Intensity (NAFDI) and the North Atlantic ocean temperature long-term variability (García et al., 2016). These islands have historically received Saharan dust as a consequence of large scale meteorological processes that involves mid-latitude waves, the NAFDI and the Saharan Heat Low (Cuevas et al., 2016). Thus, when this event takes place, the Canaries become dusty and «naturally» polluted with particulate matter (PM_10_).

Socio-economic impacts include reduced visibility which tends to affect both airports and their transport services, such as air traffic control and communication disturbance, including the closing of airports in extreme dust conditions (Dorta, 2007). However, the impacts on human health are amongst the most relevant, since respiratory pathologies, anxiety disorders, and atypical thoracic pain usually affect local population (García Carrasco et al., 2001). Other studies have reported respiratory allergic diseases leading to increased use of air liquid as a respiratory therapy as much as 600% (Belmonte et al., 2010). It has also been suggested that Saharan dust events might be related to the introduction of microbial communities, such as bacteria and viruses (Gonzalez-Martin et al., 2013).

Furthermore, summer dust events are associated with meteorological conditions that have several environmental implications. Aircraft measurements and satellite observations (Tsamalis et al., 2013) have shown that the dusty, hot and dry SAL typically expands between 1 and 5 km.a.s.l. Atmospheric soundings have shown that during intense events, the SAL occurs above 500 m.a.s.l. over Tenerife, shifting the typical inversion layer associated with the trade winds to lower altitudes and resulting in high temperatures in the forest of the Island that typically occurs between 600 and 1800 m.a.s.l. These high temperatures represent an increased risk of forest fires, whereas the shifting to low altitudes of the inversion layer is typically associated with severe pollution episodes of industrial origin in the metropolitan area, due to the emissions of the oil refinery and shipping in the harbour of Santa Cruz de Tenerife (CSIC-AEMET-UHU, 2010).

The most important sources of air pollutants in Tenerife are located along the Eastern coast of the Island (harbour and oil refinery in Santa Cruz de Tenerife, Caletillas and Granadilla power plants). The prevailing NNE trade winds coupled with the inland sea breeze blowing during daylight prompts the inlands transport of these pollutants. In Santa Cruz de Tenerife, the inland sea breeze blowing results in the inland transport of the SO_2_ plumes from the refinery and from harbour, prompting fumigations of SO_2_, sulphuric acid and ultrafine particles to the population of the city from 10 to 17 GMT (González and Rodríguez, 2013). This situation worsens under summer SAL conditions due to the concentration of air pollutants at low altitudes linked to the downward shifts of the inversion layer and to heterogeneous reactions between pollutants and Saharan dust (CSIC-AEMET-UHU, 2010).

Furthermore, the *Hospital Universitario de Canarias* and the Izaña Atmospheric Research Centre found that hospital admissions due to heart failure are associated to exposure to ultrafine particles (Domínguez-Rodríguez et al., 2011), whereas black carbon has been associated with Acute Coronary Syndrome (Domínguez-Rodríguez et al., 2016). Other studies have also observed relationships between NO_2_ and the ejection capacity of the heart (Dominguez-Rodriguez et al., 2013a) and between SO_2_ and obstructive lesions and Acute Coronary Syndrome (Domínguez-Rodríguez et al., 2013b).

A CC Agency was created in April 2009 by means of a regional law (BOC, 2009). The aim of the Agency was the promotion, encouragement, orientation and coordination of policies, initiatives and measures to reach sustainable development as well as the mitigation of and adaptation to CC. The 25th of June 2012 the Government of the Canary Islands launched a regional law intended to adopt measures to reduce public administrations expenditure in order to respond to the financial crisis. The preface of the law indicated that budget cuts were required so as to «guarantee public expenditure sustainability» within an economic crisis that obliged the public administration to accomplish the objectives of budgetary stability (BOC, 2012). As a consequence of this law, the CC Agency was shut down.

During those three years the Agency was able to develop a CC mitigation plan for the Canary Islands (Gafo-Fernández, 2009), which is currently approved, as well as a CC adaptation plan, which has not been passed into law yet (Martínez-Chamorro, 2010). However, both climate policies are currently considered to be out-dated by local climate experts (Hernández-González et al., 2016), leaving the islands without a CC adaptation strategy in a context of high vulnerability to the effects of CC (López-Díez et al., 2016). An Observatory for CC has however been established in April 2017 in Lanzarote (Canary Islands), to compensate for the lack of climate policy.

Although the negative effects of climate hazards are recognised by Tenerife administration, there is presently neither a clear adaptation strategy nor accountable institutions and actors.

## 3 Engaging relevant actors in the Tenerife Island: methodological considerations

Informing stakeholders, citizens or any other social actor is not enough in CC adaptation planning; adaptation requires deeper engagement of relevant actors including citizens (Kuik et al., 2016) from the very beginning through to the end of the process (Hernández-González and Corral, 2017). Environmental governance requires «the participation of people other than the technically qualified researchers; indeed, all the stakeholders in an issue form an ‘extended peer community’ for an effective problem-solving strategy for global environmental risks» (Funtowicz and Ravetz, 1993, p. 744). The potential benefits of societal engagement in environmental planning are various (Giering, 2011): (a) ownership of policies, (b) better decisions in terms of sustainability and the inclusion of community values, (c) environmental agencies credibility, and (d) faster implementation of sustainability planning.

Table 1 lists different CC adaptation case studies where a range of participatory methods was applied. Stocker et al. (2012) carried out a participatory process in the Rottnest Island (Australia), a small island of 19 km^2^ and 12 km long located 20 km off the cost of Perth. The island is presented as highly vulnerable to coastal erosion and floods, highlighting the need for adaptation strategies. The authors used Google Earth as a tool to engage local stakeholders and identify hotspots around the island. Schaap et al. (2013) designed a participatory process to look for adaptation strategies to adapt agricultural production to heatwaves in Flevoland (The Netherlands). Through workshops, farmers and other stakeholders were engaged in order to come up with a list of adaptation actions intended to minimise agricultural disruptions to heatwaves under different warming scenarios. Crop cover, plant and early harvesting as well as drip irrigation were proposed. Lastly, Sautier et al. (2017) applied a game-like platform to engage stakeholders in grassland-based farming systems in Aveyron (France). This game, called «Forage Rummy», aims at addressing complex farming management techniques under extreme climate conditions. Thus, farmers were able to test alternative adaptation actions, as well as assess their robustness during workshop sessions, in order to enhance real-life farming decision-making.

**Table 1:** Selected climate change adaptation participatory cases

Authors	Case study	Place	Methods	Tools	Actors	Purpose
Bojovic et al. (2015)	Agricultural adaptation	Veneto, Italy	Online questionnaire, interviews, online platform and workshop		Farmers, irrigation Boards, regional policy-makers and scientists	Suggest and assess adaptation solutions
De Stefano et al. (2017)	Wetlands management	Doñana, Spain	In-depth, semi-structured interviews, surveys, and workshops	Future scenarios	Academia, agriculture, environmental NGOs, public administration, other actors (tourism, fisheries, etc.)	Design of adaptation measures as well as their evaluation
Prober et al. (2017)	Woodland landscapes	NSW, Victoria and South Australia	Workshops		Decision-makers, graziers, farmers, community organisations, natural resources management bodies, local councils, state and federal agencies and Indigenous traditional owners	Biophysical adaptation pathways for agricultural landscapes
Roudier et al. (2014)	Crop management	Bacfassagal and Paoskoto, Senegal	Questionnaires and two-day participatory workshop	Simulation exercises	Farmers	Improving resilience of agriculture to climate shocks
Sautier et al. (2017)	Grassland-based livestock systems	Aveyron, France	Interviews, workshops and open- and closed-ended questionnaires	Pre-existing game-like platform	Farmers	Anticipate and cope with the effects of CC
Schaap et al. (2013)	Arable farming management against heatwaves	Flevoland, The Netherlands	Workshops		Farmers, sector representatives and regional policy makers	Identify adaptation measures and design an adaptation strategy
Stocker et al., 2012	Coastal adaptation	Rottnest Island, Australia	Workshop and questionnaires	Google Earth	Local authorities, staff, business community, volunteers, Indigenous representatives, state agency representatives and experts	Develop adaptation strategies

A few CC adaptation projects deployed focus groups to explore e.g., adaptation strategies for agricultural production (Dumenu and Obeng, 2016), new indicators for building resilience against diseases (Dovie et al., 2017), microfinance as means to foster adaptation for household livelihood (Fenton et al., 2017), scientific communication to facilitate the adaptation of residential buildings (Glaas et al., 2017), social issues grounding different responses to adaptation (Sapkota et al., 2016), adaptation pathways in urban water supply systems (Kingsborough et al., 2016), or adaptation frameworks for tourism destinations (Wyss et al., 2014).

**Fig. 1:**
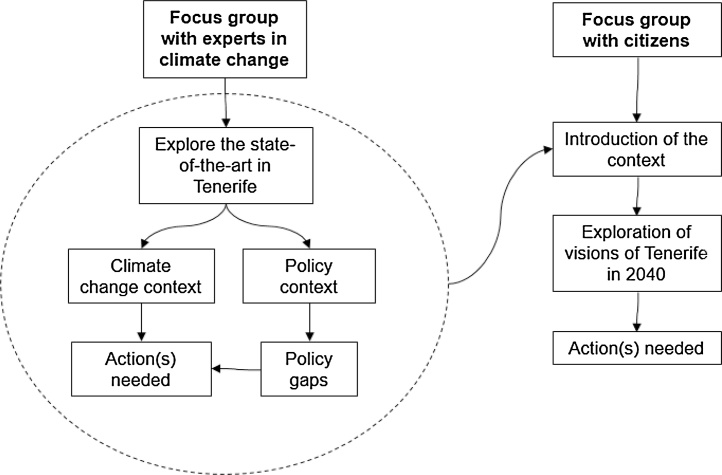
Methodological framework.

The adopted methodological framework is presented in Fig. 1. Experts in climate change were addressed first in order to outline the current scientific framing, namely how the effects of heatwaves and related hazards are addressed in existing policies as well as governance aspects of adaptation, policy gaps and organizational constraints − this context was introduced in Section 2 –. The focus groups with citizens, consisted of the exploration of visions of Tenerife in 2040, departing from the current context. Focus Groups were used here as a social research method. Bloor et al. (2001) describe this method as a form of group interviewing that pursues the collection of qualitative information to answer research questions, hence considered useful tools to learn more about a certain topic, as well as, to ascertain actors’ opinions with regards to research questions (Morgan and Krueger, 1993). They also help researchers to detect attitudes, feelings, beliefs, experiences, and reactions that would not be possible to collect by other social research methods, such as observation, one-to-one interviews or questionnaire surveys (Gibbs, 1997). We are particularly interested in the dynamics of conversations and how this changes both opinions and relationships among different actors.

**Fig. 2:**
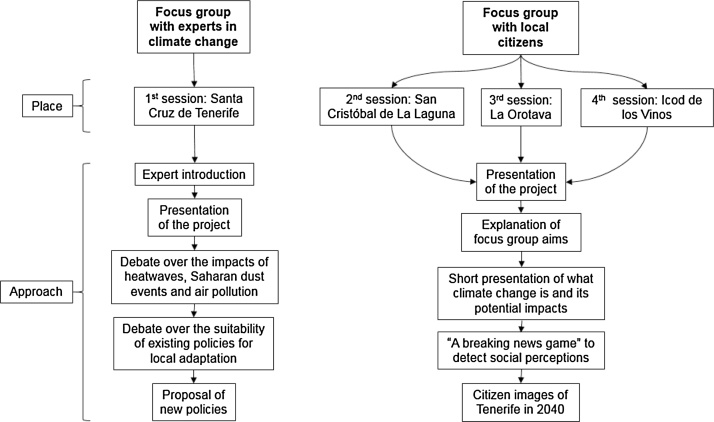
Organisation of the discussions in the focus groups.

Four focus groups (FG) were carried out in order to identify critical trends for the elaboration of adaptation visions: firstly, one session in the capital city with local experts in CC and stakeholders interested in climate policy, followed by three sessions with citizens which were held in different municipalities of the island. The FG sessions were carried out as described in Fig. 2. Two different strategies were applied to introduce the project and conduct the discussions, given the different needs of the participants.

### 3.1 Starting point: *future news*

**Fig. 3:**
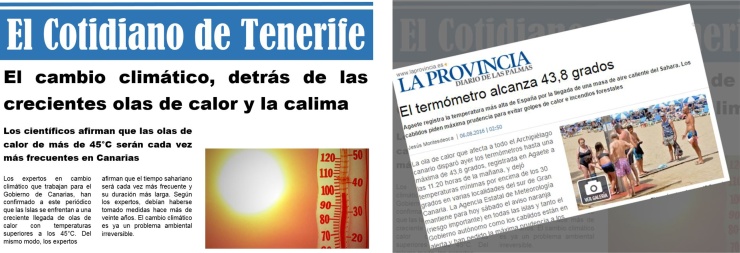
An example of breaking “future news”.

“The year is 2040, the place is Tenerife. You decide to enter in a bar to have a coffee; you pick up a newspaper, *El Cotidiano de Tenerife* [inexistent newspaper]. The newspaper contained a series of news related to CC in Tenerife [see Fig. 3 and Hernandez et al. (2017) for more details]. The headlines that you can read are the following:

− “Climate change, behind the rising of heatwaves and Saharan dust events: heatwaves surpassing 45° C will become more frequent in the Canary Islands, scientists foresee”.

− “More than 100 people hospitalized by heatstroke: a total of 117 people have had to be hospitalized during the months of July and August”.

− “The effects of heatwaves and Saharan dust collapse all Hospitals: the workers assure that it is not a punctual peak, but an increasingly frequent situation”.

− “70% of the population of Tenerife breathes polluted air: central plants, the refinery, port activities and road traffic are the main cause”.

− “Wine production at risk due to climate change: winegrowers face a 60% loss in wine production because of rising heat”.

− “Tenerife runs out of potatoes for the first time in history: the growing heat is responsible for the loss of all potato production on the island”.

After exploring participants’ views about the “future news”, a number of related real breaking news were showed to participants for them to ‘compare’ the hypothetical 2040 scenario with what is already happening in the island. This exercise was useful for participants to raise awareness of possibly already occurring ‘extreme’ situations, which are in need of urgent action.

All participants could *flip through* a print-out of the *newspaper,* being subsequently asked about how they thought they could be affected under these futuristic snapshots, and whether these could be considered extreme.

During the second part, participants were asked to imagine how would Tenerife look like in 2040, since the increasing concentration of greenhouse gases (GHG) has, according to WMO (2016), marked the beginning of a new climatic era. Participants were subsequently asked to make specific proposals that would allow Tenerife to be on the path of adaptation to potential increasing heatwaves as well as its expected impacts (see Section 2). A number of 2040 visions were produced.

### 3.2 Climate adaptation dialogues

The first FG session took place in Santa Cruz de Tenerife, which is the most populous municipality of the Island, with almost 204,000 inhabitants (ISTAC, 2017a). Santa Cruz de Tenerife belongs to the group of municipalities that have signed the Covenant of Mayors (2017). 19 people were invited to attend the event (6 climate experts from the University of La Laguna, 3 from the Government of the Canary Islands, 2 from the Council of Tenerife – including the Covenant of Mayors coordinator –, 2 from the Izaña Atmospheric Research Centre, 2 from local trade unions, and 1 from the Chamber of Commerce, environmental NGO, Institute of Volcanology and the Council of Gran Canaria). All participants were either experts in CC or stakeholders involved in disaster risk management.

The second session was held in San Cristóbal de La Laguna. It is a municipality that belongs to the metropolitan area of Tenerife. It is the second most populous municipality of the Island, with more than 152,000 inhabitants (ISTAC, 2017a). San Cristóbal de La Laguna also belongs to the group of municipalities that signed the Covenant of Mayors (Covenant of Mayors, 2017). Citizens were invited through an open call using social media and local newspapers. Fifteen people attended the event. The educational level of the participants was high. All of them had a University degree or post-graduate education.

The third session was set in the municipality of La Orotava. This municipality has a population of about 41,000 inhabitants (ISTAC, 2017a). As in San Cristóbal de La Laguna, an open invitation call of citizens was organised. 18 people attended the event. The educational level of the participants was, generally, high. However, unlike the second session, this one was attended by people who did not have CC expertise, albeit interested in the subject.

The fourth session took place in the municipality of Icod de los Vinos, which has a more rural character. Its population is almost 23,000 inhabitants (ISTAC, 2017a). The same procedure was used to invite local citizens. 10 people attended the event and actively participated in the debate. The main feature of this last session was that, with some exceptions, most of the people were not experts. This smaller number of participants allowed more thoroughly exploration of concrete measures to adapt to the heat, Saharan dust events and air pollution.

**Fig. 4:**
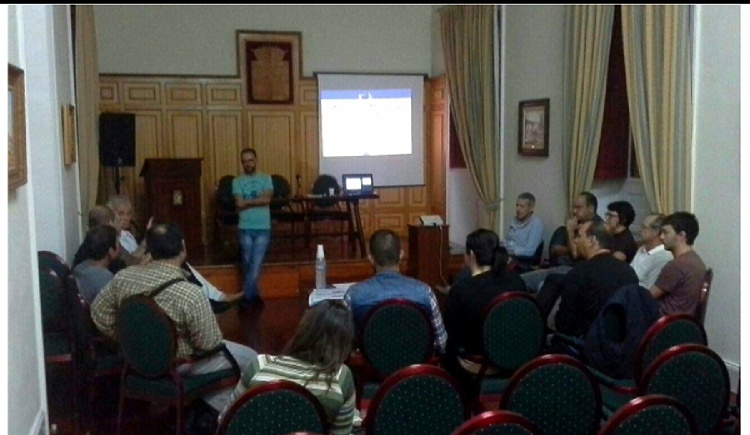
Focus group session in La Orotava.

The next sections summarise and discuss the views and insights given by the experts, stakeholders and citizens who attended the four FG sessions – Fig. 4 illustrates one of the sessions.

## 4 Results and discussion

During the FG sessions the concept of resilience was explored, with the following meanings: energy use reduction, increasing food production sovereignty, and sustainable water resources management. In other words, participants sought to position their concerns and insights into a broader frame of governance of human societies and natural systems. The key issues that interested the dialogues about CC adaptation were all related to governance issues, including resources sovereignty, articulated policies across sectors, respect for the island’s carrying capacity, maladaptation strategies to CC, and inequality. They are all seen as hindering factors to enact useful strategies and action to protect humans and nature in Tenerife. In addition, the lack of symbolic institutions that address CC is considered another caveat towards serious action.

### 4.1 Sovereignty: a key issue for the participants

Participants raised the need for energy, food and water sovereignty, addressing the problem not only from the supply side, but also through the management of demand.

#### 4.1.1 Energy sovereignty

The external dependency on fossil fuels was a recurring issue in all discussions; only 1.5% of primary energy production comes from renewable energies in the Canary Islands (CEICC, 2016). Thus, according to the participants, the external energy dependency leaves the Islands with no chance of meeting their basic energy needs in face of fossil fuel price shocks (see Table 2). Therefore, this dependency is perceived as more important than other threats like CC, as stated by a participant in La Orotava: «*I think that the energy collapse is almost more pressing, in terms of temporality, than climate change*» (in Hernandez et al., 2017).

**Table 2:** Citizen statements regarding energy dependency and actions needed.

C1. «I think of Tenerife in 2040 and the oil issue comes to my mind. I don’t know what the oil price will be in 2040 but I think that the Canaries are very sensitive. I’m not a specialist, but I guess that there’re things that can be solved with renewable energies if we invest resources as soon as possible». (FG held in San Cristóbal de La Laguna).
C2. «In Germany, at least as far as I have understood, zero dependency from fossil fuels by 2060 is expected for both oil and coal. So why is Germany, located in the North, already raising such issues when we still seem to be afraid of change? And say, come on, let's change this». (FG held in La Orotava).
C3. «I think that, if the Government wants, we’re in a position of supplying the Canary Islands with renewable energies by 2040». (FG held in La Orotava).
C4. «It is clear that a change in the energy model is urgent and possible. Apart from that technological change and that energy model, a reduction of energy consumption is also important, because it is clear that, e.g., today washing machines are more efficient, but now 4 washing machines are sold instead of 1 sold before. (…) The idea that I want to raise is that a change in the energy model and technology in general is super important, it being possible and necessary, but it has to be accompanied by consciousness and reduction of consumption or else there is no useful technology». (FG held in La Orotava).
C5. «We should create a budget that specifically integrates climate change and sustainability. That is, where do we get the money from? By charging more all those polluting activities. For example, private car use». (FG held in San Cristóbal de La Laguna).
C6. «I would add an idea regarding the creation and the real implementation of sustainable mobility plans: public transport, dissuasive private motorized transport measures, and encourage cycling and walking. And what concerns me the most: the implementation. Because there are plans already». (FG held in San Cristóbal de La Laguna).

Furthermore, an energy system based on fossil fuels keeps the emissions of GHG growing; in fact, GHG emissions have grown 45% since 1990 in the Canary Islands (CEICC, 2016). The participants insisted on the necessity to increase the production of renewable energies in the energy mix of the islands (in general) and Tenerife (in particular) (see comments C1, C2, and C3 in Table 2), passing through a greater investment in research and development, especially linked to the promotion of renewable energy (see comment C4 in Table 2). Furthermore, energy consumption should also be reduced (see also comment C4 in Table 2).

The need to introduce green taxation was proposed, based on dissuasive wasteful uses of energy, which would penalise higher consumption (see comment C5 in Table 2). Implementation of sustainable mobility plans by reducing mobility rates, was also suggested (see comment C6 in Table 2). The use of cars should be discouraged, whilst alternative modes of transport should be promoted.

#### 4.1.2 Food sovereignty

The external dependency on food products has been a recurring issue in all discussions. In fact, the level of food self-sufficiency in the Islands is considered low and decreasing (Godenau and Nuez-Yáñez, 2013). The participants exposed concern on the low chance of meeting their basic goods needs in the face of possible external shocks (peak prices, etc.): «*imagine there is an international conflict. We depend exclusively on what comes from abroad by sea and air. There are economic studies indicating that we would have provisions for 3* *days, on the fourth day we would kill each other if there was no external supply*» (FG held in La Orotava; see Hernandez et al., 2017).

A strong debate focused on agricultural protection policies and their potential effects on basic goods’ prices. In this regard, a participant stated in San Cristóbal de La Laguna: «*I believe that food sovereignty and a clear commitment to agroecology is essential for the Canary Islands*» (in Hernandez et al., 2017). Participants agreed over the need to increase local agricultural production based on sustainable ways, especially to improve soil quality.

#### 4.1.3 Sustainable water use

The participants were concerned about how to promote, in line with the Water Framework Directive (WFD), a «sustainable water use based on a long-term protection of available water resources» (OJEC, 2000, p. L 327/5). This concern might be seen in the following statement given by a citizen in Icod de los Vinos: «*the population needs 30* *L of water a day and with the system we have now,infiltration galleries are depleting. (…) There’s already an alarm highlighting that there’s not enough. (…) Here the wells have reached the water table. We’re in the middle of the sea and the sea obviously penetrates the inner island and there will come a time when you start drilling, looking for water, you’ll reach the water table and what you find is salt water*» (in Hernandez et al., 2017).

According to the participants, the main objective should be water storage and use reduction policies in order to adjust water demand to the biophysical capacities of natural water in Tenerife (as implicitly stated in the comments given in Table 3).

**Table 3:** Citizen statements regarding the need for sustainable water use.

C1. «One issue that I think is important is water management. Yes, because of my local climate experience, when a Saharan dust event occurs, there is no rain. This can affect water resources that are already a big problem». (FG held in La Orotava).
C2. «You all should check the energy and water consumption statistics of 15 or 20 years ago so that you know what it is that they [politicians] call sustainable development». (FG held in San Cristóbal de La Laguna).
C3. «We receive millions of tourists a year. My question is: what do we really want for 2040: socioeconomic activity and bring water from other places?» (FG held in La Orotava).
C4. «I have a cistern of 90 m that was made in the year 1805. It fills up in a day of rain. And I water all my plants using the water from the cistern. I do not drink it, of course, I cannot drink it because it is somewhat contaminated, but I water with it. A normal rainy day, with this last rain, I fill a deposit of 90,000 L. Why architects don’t demand, when they build a house, to make a cistern to collect rainwater? It’s such a simple and easy saving. We’re talking about 90,000 L of water». (FG held in La Orotava).

### 4.2 *Maladaptation* to climate change and green infrastructure

Participants pointed out that both the use of sea water desalination (comments C1 and C2 in Table 4) to supply water under a scenario of drought or the use of air-conditioning under heatwaves events (comments C3 and C4 in Table 4) may imply *maladaptation* and how some strategies lead instead to increasing use of energy.

**Table 4:** Citizen statements regarding the need to avoid maladaptation and encourage green infrastructure.

C1. «If the forest finishes, drinking water runs out, and it’ll have to be taken from the sea. Desalinating water, making it drinkable is very expensive, requires a lot of energy». (FG held in Icod de los Vinos).
C2. «Among other things, if precipitation decreases, desalination will be required. This implies an energy expenditure that has to be paid as well as air conditioning. These are serious problems». (FG held in La Orotava).
C3. «Unless we change energy production, turning on the air conditioning means increasing the pollution a lot due to the power plants». (FG held in San Cristóbal de La Laguna).
C4. «I think, I don’t have the data but surely, the consumption of air conditioning in the buildings, both public and private, has skyrocketed. This requires a rethinking of architecture and urbanism». (FG held in La Orotava).
C5. «The urban landscape by law was approved. It was pioneer regulating the distribution of surface on the roofs, where there must be a surface dedicated to renewable energies and there must be a percentage of landscaped area». (FG held in La Orotava).
C6. «I would propose gardens on the roofs to generate microclimates, more shade». (FG held in San Cristóbal de La Laguna).

The maladaptation discourse lead participants to talk about renewable energy (comment C3 in Table 4) and bioclimatic housing for new buildings (comment C4 in Table 4): proper insulation maintains good in-door living conditions, reducing the energy cost of additional air conditioning.

Participants also suggested that green areas and green infrastructure should be extended, not only to generate shade, but also to improve air quality: «*I would implement ecological principles in cities; they could be covered with green. Lots of more trees and vegetation everywhere. That does help against heatwaves. The air would be cooler due to all those green areas. Air pollution would improve too, because trees consume CO*_*2*_*. In addition, the more green areas the more permeable zones we’d have in the cities, since land absorb water. We are talking about super powerful drainage systems. And it would avoid flooding, for example. It's not just a matter of temperature, I think*» (from FG held in San Cristóbal de La Laguna; see Hernandez et al., 2017). Such green infrastructure could consist of e.g. urban parks and afforestation programmes and/or private small green areas in roofs (see comments C5 and C6 in Table 4).

### 4.3 Coherent environmental governance

Good environmental governance understood as the combination of citizen participation during both the assessment and post-assessment processes of environmental planning, is necessary to empower participants to check the social robustness of environmental planning (Hernández-González and Corral, 2017). Furthermore, good climate governance also requires stable institutions able to work in climate policy regardless of electoral cycles or political changes (EEA, 2012).

According to the participants, local environmental governance is an issue in the island. See for example the statement given in La Orotava: «*the energy policy we have in the Canary Islands is regrettable. Irrespectively of the political party. The issue of alternative energies is an insult to all humanity. I haven’t been a politician, sometimes I’m tempted, because there is so much waste of resources, money and ideas; that you say: is there no one who has a little common sense? The Canary Islands must be totally self-sufficient in energy, and it can be*» (in Hernandez et al., 2017).

One of the main problems highlighted was the non-compliance with environmental protection laws and their monitoring phases (see comment C1 in Table 5). Contradictory objectives in public environmental policies are in need of attention (e.g., whereas the Department for Transport of the Council of Tenerife aims at reducing car usage, the Department for Infrastructure pursues building new roadways); greater coordination among Public Administrations to conciliate incoherent disparate policy objectives is urgent.

**Table 5:** Citizens’ statements regarding the need for better governance.

C1. «In Spain we have the habit of writing public policies on paper, but public policies are made all around the world by committing resources and monitoring mechanisms to see if the objectives are met or not». (FG held in San Cristóbal de La Laguna).
C2. «I was thinking that we really need to have citizen power, because political power is pursuing other interests, which are not the general interests of all». (FG held in La Orotava).
C3. «For me, citizen commitment is as important as public policy. Because if we compare ourselves with other territories, that territories are more advanced in this type of issues, for example, Catalonia. And one wonders why? Because society has another way of living public life there. We should ask ourselves here, how do we live it? How do we delegate problems? As the fellow said, for me, this is how we should move forward, that we would be able to decide. It's commitment as well. We simply have to get everyone involved». (FG held in San Cristóbal de La Laguna).

Participants also argued for citizens’ empowerment (see comment C2 in Table 5) in decision-making through genuine participatory processes, i.e. processes that are not based on information and consultation, but on the inclusion of citizens (see comment C3 in Table 5) from the first steps of land-use planning to the final follow-up processes. Lastly, the need to reopen the Canary Islands Agency for Sustainable Development and Climate Change was seen as a key institutional requirement to foster coordinated planning and action. This Agency should be the unifying agent of sustainability objectives among all public and private administrations.

The existing competition for water resources between the tourism and the agricultural sectors raised another important issue which is the competing vested interests long established in the Canary Islands.

### 4.4 Carrying capacity: tourism sector

Tourism was featured in all discussions as a key element. For example, can Tenerife be resilient depending on the tourism sector? Is sustainable tourism possible? Could 6.4 million tourists’ (ISTAC, 2016) fly to Tenerife in the post-oil era? Is anyone planning for this scenario? These were key questions that emerged during the debates, which had no answer from any participant that attended the FG. The following statement, given in La Orotava, summarises this general concern: «*most likely we’ll reach 14 (millions of tourists per year). But, unintentionally, we need to move more than 90% of tourists by plane. It is clear that this will not be possible by means of alternative energy. You can do other things, but really, planes, according to the studies that I’ve read, that’s impossible. (…) Due to the increasing temperatures, fewer tourists may access the tourist areas. As we’re living almost exclusively from that, the future looks pretty dark to me*» (in Hernandez et al., 2017).

Some ideas were proposed such as an eco-tax which revenues could be used to improve the island's environment and its resilience. Secondly, the possibility of introducing a population/tourist carrying capacity in the Islands was also discussed. These two ideas are illustrated in the next statement given in La Orotava: «*I had two actions, two proposals for actions. One related to tourism; it’s the carrying capacity of visitors. I raised this idea many years ago in a worldwide site that I almost got killed by the tourism sector. It was the possibility that the tourist who has to come to the Canary Islands pay a fee. I considered the Canary Islands so beautiful, so precious, especially related to the world of National Parks that the tourist who wants to come to the Canaries has to pay a tax for visiting the Islands. (…) I insist, given the trend of tourists in the Canaries, we must limit the carrying capacity. The Canaries cannot resist 14 million, with all respect. (…) what we cannot have here is this overpopulated island that cannot stand more people anymore*» (in Hernandez et al., 2017).

These FG illustrated the difficulties of discussing sustainability in the tourism sector, especially when the strongly implemented hospitality industry depends on the arrival of millions of tourists a year, who travel by airplane, the most intensive fossil fuel energy consumer means of transport (Sanz et al., 2014).

### 4.5 Inequality

Participants recognised that poverty and inequality increase vulnerability of persons with fewer resources (see comment C1 and C2 in Table 6) who less chances to deal with extreme weather events than wealthier people (Sen, 2000). In fact, severe poverty has increased in the Islands by 66% during the economic crisis, whereas the number of in-work poverty (working poor) has also increased (Padrón Marrero et al., 2016).

**Table 6:** Citizens’ statements regarding the need to reduce poverty.

C1. «Well, if we go out under heatwave conditions… I find it difficult to adapt to this temperature. And to all those who work as farmers will be quite affected. For example, if you work in an office you will feel the heat, but you have air conditioning. That you have to pay more? Well, we get a salary. But when you are out there, in the farms, it is more complicated. It will be the working class the ones that will be most affected». (FG held in San Cristóbal de La Laguna).
C2. «I would think that there are people who do not have the means I have to bear heatwaves: turn on the air conditioning or go to the coast or the mountain». (FG held in San Cristóbal de La Laguna).

### 4.6 Similarities and contrasts in citizens’ insights

The researchers presented the state-of-the-art in the FG, focusing on the effects of heatwaves, Saharan dust events and the possible relations with air pollution, pointing out the likely increase of these threats in the future. However, the citizens argued that the governance issue is not just adapting to climatic threats, but rather to make the island resilient to any external shocks, including climatic, economic or political. This is an interesting contrast among CC experts and those who participated as citizens.

Furthermore, whereas governmental experts in CC tended to propose the reinforcement of governmental institutions to tackle heatwaves in particular, and CC in general, several citizens tended to see the governmental institutions as part of the problem – see quote C2 in Table 5.

With regards to the educational level, citizens with higher education level tended to think about the problem systemically, proposing solutions to the problem by acting on its underlying causes; many of those proposals would imply significant policy changes. In contrast, the participants with lower educational levels tended to be more practical, i.e. they would rather focus on very concrete actions to solve possible CC impacts.

## 5 Conclusions

The case study presented for Tenerife focuses on heatwaves, Saharan dust events, and local air pollution episodes, which may occur separately or simultaneously. These three hazards produce negative effects in local ecosystems and humans. Finding strategies to adapt to these events and their impacts is crucial in order to reduce morbi-mortality and environmental downside effects in Tenerife.

This study brought insights into relevant institutional arrangements, social actors and desirable strategies to organise and deploy meaningful action against CC events in Tenerife. The participants of the focus groups discussed resilience as the main governance issue rather than focusing on the lack of adaptation strategies to heatwaves or other extreme weather events. In response to the researchers’ framing focused on the threats of those hazards, participants pointed out that making the island resilient to external shocks, be it climatic, economic or political was the issue that needed to be addressed.

We adopt here the IPCC (2014) definition of resilience, i.e. «the capacity of social, economic, and environmental systems to cope with a hazardous event or trend disturbance, responding or reorganizing in ways that maintain their essential function, identity, and structure, while also maintaining the capacity for adaptation, learning, and transformation» (IPCC, 2014, p. 1772).

Hence, resilience appears to be the response that institutions and societal actors need to aim for when designing adaptation strategies to tame CC. Participants of this engagement exercise have proposed and critically examined desirable and feasible scenarios and actions within this new frame, developing meanings of resilience in the context of the island. In this new frame, the meanings of resilience for Tenerife were expressed in the following interlinked ways: 1) a sovereignty issue, 2) maladaptation avoidance, 3) environmental governance coherence, 4) sustainability of tourism, and 5) reduction of inequalities.

Sovereignty meant energy use reduction, increasing independency of food production, good water resources management respectful of the insular biophysical limits, etc. In other words, participants sought to position their concerns and insights into a broader frame of governance of human societies and natural systems. This would imply a societal and governmental commitment towards increasing sovereignty levels. Maladaptation avoidance is clearly linked not only to sovereignty but also to coherent environmental governance. The nexus water-energy-food is relevant here as mitigation measures of CC can lead to increased stresses in energy resources: e.g. more energy for cooling, more desalination of water, etc. The tourism sector is by far the dimension with larger uncertainties. For example, can Tenerife be resilient depending on the tourism sector? Is there something like sustainable tourism? Could 6.4 million tourists’ fly to Tenerife in the post-oil era? Is anyone planning this scenario? These questions emerged during the debates, with no clear answers. Last but not least, inequality is an issue in Tenerife: population below relative poverty in Tenerife reaches almost 24% (ISTAC, 2017b). This has a relevant implication for the local context and requires deep transformations of societal organisation.

### 5.1 Final remarks

The CC adaptation frame seems to be too narrow, especially in contexts characterised by insularity and other specificities. Indeed, the key issues that interested the dialogues about CC adaptation in a way or the other were all related to governance issues, including resources dependencies, unarticulated policies across sectors, disrespect for the island’s carrying capacity, maladaptation strategies to CC, and inequality. They are all seen as hindering factors to enact useful strategies and action to protect humans and nature in Tenerife. In addition, the lack of symbolic institutions that lead action is considered another caveat towards serious action. The framing of resilience developed during the participatory process seems to be the desirable strategy to enact necessary transformation about those hindering factors.

Global phenomena do not manifest equally around the planet, so any attempt to deal with CC issues needs to be situated. Involving relevant actors, including citizens means mobilisation of relevant types of knowledge to address in a hopefully lesser contended way the challenges that a changing climate of natural, social, economical and political nature bring today. The futuring angle of the methodology applied in this study was an interesting trigger to wonder about framings, uncertainties, expectations and imaginaries about strategy and action. Finally, we argue that dialogues about CC challenges are not only desirable but actually should be the pillar of any strategic development to address locally what is heralded as a global phenomena, not least because it is unimaginable that any thoughtful, feasible or desirable action can be taken without situated knowledge inputs in “insular” territories.
